# Core Alzheimer’s disease cerebrospinal fluid biomarker assays are not affected by aspiration or gravity drip extraction methods

**DOI:** 10.1186/s13195-021-00812-9

**Published:** 2021-04-16

**Authors:** James D. Doecke, Cindy Francois, Christopher J. Fowler, Erik Stoops, Pierrick Bourgeat, Stephanie R. Rainey-Smith, Qiao-Xin Li, Colin L. Masters, Ralph N. Martins, Victor L. Villemagne, Steven J. Collins, Hugo Marcel Vanderstichele

**Affiliations:** 1 The Australian e-Health Research Centre, CSIRO, Brisbane, QLD Australia; 2 ADx NeuroSciences, Ghent, Belgium; 3 University of Melbourne, Melbourne, VIC Australia; 4 Australian Alzheimer’s Research Foundation, Perth, WA Australia; 5 Centre for Healthy Ageing, Health Futures Institute, Murdoch University, Murdoch, WA Australia; 6 Edith Cowan University, Perth, WA Australia; 7 Department of Molecular Imaging & Therapy, Austin Health, Heidelberg, VIC Australia; 8 Department of Psychiatry, University of Pittsburgh, Pittsburgh, PA USA; 9 Biomarkable, Ghent, Belgium

**Keywords:** Cerebrospinal fluid, Amyloid beta, Alzheimer’s disease, Biomarkers, Collection, Concordance

## Abstract

**Background:**

CSF biomarkers are well-established for routine clinical use, yet a paucity of comparative assessment exists regarding CSF extraction methods during lumbar puncture. Here, we compare in detail biomarker profiles in CSF extracted using either gravity drip or aspiration.

**Methods:**

Biomarkers for β-amyloidopathy (Aβ1–42, Aβ1–40), tauopathy (total tau), or synapse pathology (BACE1, Neurogranin Trunc-p75, α-synuclein) were assessed between gravity or aspiration extraction methods in a sub-population of the Australian Imaging, Biomarkers and Lifestyle (AIBL) study (cognitively normal, *N* = 36; mild cognitive impairment, *N* = 8; Alzheimer’s disease, *N* = 6).

**Results:**

High biomarker concordance between extraction methods was seen (concordance correlation > 0.85). Passing Bablock regression defined low beta coefficients indicating high scalability.

**Conclusions:**

Levels of these commonly assessed CSF biomarkers are not influenced by extraction method. Results of this study should be incorporated into new consensus guidelines for CSF collection, storage, and analysis of biomarkers.

## Background

Dementia is a syndrome with a complex pathophysiology, characterised by a heterogeneous group of clinical features and pathological hallmarks (e.g., β-amyloidopathy, tauopathy, synapse loss, oxidative stress, inflammation) [1]. In particular, Alzheimer’s disease (AD) has a long pre-clinical phase (20–30 years) [2] with pathological detection requiring visualisation of on-going amyloidopathy and tauopathy in brains of affected subjects, preferably in an early phase of the disease [3].

Pathological diagnosis can be achieved by regulatory approved β-amyloid (Aβ)-positron emission tomography (PET) and Tau-PET imaging methods [4]. PET technology is considered a minimally invasive method, with little to no complications or risks for the subject. Harmonisation is required between tracers and interpretations performed by the individual investigators. Furthermore, each tracer utilises a different brain region as negative control and each has a different specificity towards Aβ-oligomers [5]. Only visual interpretation of scans by trained physicians is presently approved by the Food and Drug Administration (FDA) [6]. The method is expensive and requires highly specialised instrumentation, training, and logistics [7]. Results are used by clinicians to estimate Aβ-neuritic plaque density or tangle load (FDA Tauvid [8]) in adult patients with cognitive impairment who are being evaluated for AD and other causes of cognitive decline.

Cerebrospinal fluid (CSF), which surrounds the brain and the spinal cord, is considered as a “mirror” of the brain [9]. CSF collection is a minimally invasive procedure, which is safe when performed with care and appropriate precautions [10, 11]. Changes in CSF protein biomarker profiles, when measured accurately, demonstrate abnormalities at least 20 years prior to the expected age of onset in dominantly inherited AD pedigrees [12]; earlier than can currently be detected with imaging modalities [13]. In the last two decades, CSF diagnosis of AD has focused especially on the quantification of proteins that have been identified in plaques and tangles, such as Aβ1–42 and phospho-tau [14, 15]. More recently, other CSF proteins (e.g., alpha-synuclein [α-synuclein], Neurogranin [Ng], beta-site amyloid precursor protein cleaving enzyme 1[BACE1], neuropentraxin2, neurofilament [NFL]) have revealed the presence of co-pathologies/co-morbidities in the brain, such as Lewy bodies or loss of synapses. The latter can help to predict the rate of future cognitive decline and will likely provide tools for better stratification of individuals for inclusion in clinical trials. At the regulatory level, efforts are on-going in Europe (European Medicines Agency (EMA)) and the USA (FDA) to qualify CSF proteins for inclusion in clinical trials [16, 17].

CSF AD biomarker analysis, especially for inter-laboratory comparisons, has previously been hampered by variabilities across pre-analytical handling, assay design, and laboratory performance, exacerbated by the absence of consensus on how to collect, process, and store CSF [18]. Consequently, considerable differences in absolute concentrations of AD biomarkers are reported by different centers using the same assay, leading to non-uniform cut-off values for an identical context of use. These difficulties have been mitigated by (i) the establishment of the Alzheimer’s Association quality control program which has documented a continuous improvement of assay performance by dedicated vendors of the immunoassays [19], (ii) the release of certified reference methods for analysis of CSF Aβ1–42 [20] (no reference materials are available yet for the other CSF proteins), and (iii) the introduction of automated biofluid analysis platforms to reduce inter/intra laboratory variation [21]. These achievements have allowed diagnostic laboratories to establish internal operator training programs and provide a tool to the assay vendors to harmonise their results with the current best practice in the field. However, accurate quantification of CSF proteins still requires extensive standardisation of sample analysis, as well as standard operating procedures for collection and storage of CSF [22].

Several guidance papers have been published with the ultimate goal of generating a consensus on how to handle CSF sample for analysis of Aβ proteins [18, 22, 23]. Except for the most recent guidance [24] (Hansson et al. The Alzheimer’s association international guidelines for handling of cerebrospinal fluid for routine clinical measurements of amyloid β and tau. Alz Dementia. Submitted), most recommendations were based on expert opinion rather than experimental evidence. A key issue not considered in detail in the guidance documents is the methodology for CSF collection through lumbar puncture (LP). During LP, CSF can be collected by allowing it to drip into the collection tube (gravity drip) or by aspiration with a syringe (aspiration). Proponents of gravity drip maintain that when a syringe is used to aspirate CSF, the extra surface area of the syringe (even when it is polypropylene) may adsorb analytes and thus influence assay results, while others believe that if a suitable polypropylene syringe is used, the resulting assays for AD biomarkers will be unaffected [25]. The method of CSF collection is an important issue because taking volumes of CSF greater than 10 mL by gravity drip is time consuming and can be uncomfortable for patients. Time taken could be an impediment to routine CSF sampling if higher throughput is desired for both diagnosis and monitoring of AD. Standardised guidelines are published for LP CSF extraction [26]; however, these are yet to provide evidence as to consistency of results post different extraction methods.

To objectively investigate potential effects of CSF collection methodology through LP when assaying AD biomarker concentrations, we provide a detailed comparison of gravity drip collection with aspiration using a polypropylene syringe during the same collection for each subject. All other pre-analytical and analytical aspects of the procedures for sample analysis were identical. We hypothesised that analyte concentrations would be unaltered by extraction method, not only for Aβ and tau proteins, but also for synapse proteins.

## Methodology

### Participant information

CSF samples from 50 participants of the Australian Imaging, Biomarkers and Lifestyle (AIBL) study were collected using aspiration then gravity drip methods during the same LP visit. Participants were deemed either cognitively normal (CN) (*N* = 36; 70%), to have mild cognitive impairment (MCI; *N* = 8; 15%) or to have AD (*N* = 6; 15%) after clinical and neuropsychological assessments, conducted as previously described [27]. Clinical assessment was taken within 6 months of CSF collection. Data for clinical parameters such as Mini-Mental State Exam (MMSE), Clinical Dementia Rating (CDR) score, the AIBL Pre-clinical Alzheimer’s Cognitive Composite (AIBL-PACC), and Apolipoprotein E ε4 (*APOE ε4*) allele status were available for all participants. All data represented in this study are cross sectional.

### Lumbar puncture

CSF was collected by LP, in the morning, from overnight fasted participants, using protocols described in detail elsewhere [28]. Briefly, the LPs were performed with the subjects in a sitting position, using a Temena (Polymedic®, EU, tamena.com) spinal needle micro-tip (22/27G × 103 mm; CAT 21922–27), or a RapID set pencil point spinal needle (25G; Smiths Medical ASD, Inc., Keene, NH, USA) if there was difficulty with the fine needle. Initially, up to 6 mL of CSF was aspirated for routine microbiological/biochemical assessment and other concurrent studies, then 8 mL of CSF was collected by gravity drip into a 15 mL polypropylene tube (Greiner Bio-One188271, Fisher Scientific, Goteborg, Sweden), and placed onto wet ice. After gravity collection, a polypropylene syringe (BD, North Ryde, NSW, Australia) was then used to aspirate 2 mL of CSF, which was then transferred to a second Greiner Bio-One188271 polypropylene tube, on wet ice. Samples were processed within 1 h by centrifugation (2000 × g, 4 °C, for 10 min) and the supernatant transferred to a new Greiner Bio-One188271 polypropylene tube before being aliquoted in 300 μL volumes into Nunc Cryobank polypropylene tubes (NUN374088, Thermo Fisher, MA, USA). Samples were stored in liquid nitrogen vapour tanks and only thawed once, immediately before analysis. Prior to thawing, CSF was shipped on dry ice to ADx NeuroSciences and stored at − 80 °C until the biomarker analysis. The range of time taken to collect the CSF by gravity was 10–15 min and the range for aspiration was 0.5–1 min. Parameters of the sample collection and adverse incidents have been reported previously [28].

### Biomarker assay

Samples from the 50 participants were tested for six analytes in the facilities of ADx NeuroSciences: α-synuclein, Aβ1–42 (herein reported as Aβ42), Aβ1–40 (herein reported as Aβ40), total tau, BACE-1 and Ng (trunc P75) (Assay details are described in Table 1). In parallel, all samples were verified for blood contamination by testing for haemoglobin (Hb) content using an in-house developed Hb-assay [29]. The latter is required to allow a more accurate interpretation of the α-synuclein concentrations obtained [30]. Samples from one subject were added onto each ELISA plate, aimed at reducing inter-plate variability. Furthermore, for Aβ40 analysis the samples were pre-diluted 21-fold with the sample diluent into a predilution plate that was treated with the same diluent for 30 min (as described previously in [31]). For all other 5 analytes the samples were directly added into the antibody-coated plates for analysis. The reported concentrations were based on duplicate sample measurements for all six analytes except for Aβ42, Aβ40, total tau, BACE-1, wherein respectively 7, 8, 1, and 2 samples were tested singly (due to a technical error or too low remaining volumes). All samples had a value within the measuring range as defined by the provided calibrators.

**Table 1:** Assay characteristics for each individual biomarker

Analyte			Amyloid β 1–40	Amyloid β 1–42	Total Tau	Neurogranin Trunc P75	BACE-1	α-synuclein
**Technology**			Sandwich ELISA (colorimetric)					
**Vendor**			Euroimmun	Euroimmun	Euroimmun	Euroimmun	Euroimmun	Euroimmun
**Product code**			EQ 6511–9601-L	EQ 6521–9601-L	EQ 6531–9601-L	EQ 6551– 9601-L	EQ 6541–9601-L	EQ 6545–9601-L
**Assay design**	**Capture mAb**		ADx103 (2G3)	ADx102 (21F12)	ADx201	ADx451 (ADxNGCT1)	ADx401 (5G7)	ADx301
	**Detector mAb**		ADx101 (3D6)	ADx101 (3D6)	ADx215	ADx403 (ADxNGCI2)	ADx402 (10B8F1)	ADx302
	**Sample Incubation**	Sample volume (μL)	15	15	25	15	15	25
		Detector volume (μL)	100	100	100	100	100	100
		Incubation time (h)	4	4	4	4	4	4
		Incubation temperature (°C)	18–25	18–25	18–25	18–25	18–25	18–25
		Sample dilution factor (1:x)	21	No dilution	No dilution	No dilution	No dilution	No dilution
	**Run validation data**	%CV QC samples (n = 3; min-max)	9.6–14.5	4.5–20.8	9.5–10.2	3.6–6.4	5.9–12.4	4.3–5.8
		%CV Kit controls (*n* = 2; min-max)	6.0–9.3	2.6–5.0	3,3–7.7	4.1–4.3	4.1–8.1	3.8–3.9
		Intra-assay CV (%) (*n* = 2; min-max)	0.0–15.3	0.0–23.6	0.0–17.3	0.0–12.9	0.0–8.8	0.0–12.7
	**Calibrator**	Calibrator range (pg/mL)	54–986	81–1724	56–1552	53–1799	238–11,212	150–5988
	**Sample concentration range (pg/mL)**	p5	3785	188	251	154	1098	1226
		p25	5933	383	342	229	1647	1742
		p50	8500	589	405	350	2091	2311
		p75	10,529	882	529	472	2904	2952
		p95	13,542	1272	812	745	3583	4190
		Minimum	2431	92	186	115	940	1085
		Median	8501	589	405	349.5	2091.5	2311.5
		Maximum	18,307	1592	922	1194	5172	5388

*Abbreviations*: *Aβ* amyloid beta, *mAb* monoclonal antibody, *p5* percentile 5, *pg/mL* picograms/milliltre, *CV* coefficient of variation, *μL* microlitre

Different levels of run-validation acceptance criteria were integrated in the test procedure. For each test run, both kit control samples (Positive Control 1 and 2, prepared using lyophilized calibrators) were within the acceptance range as described on the certificate of analysis of the kit lot used. Criteria for blank value (OD < 0.100) and calibrator curves (OD highest calibrator > 1.2) were approved. In addition, three “in-house” QC samples (QC1, QC2, QC3) were included in each assay run for assessment of test run performance. The composition of the QC samples is based upon neat CSF obtained from a commercial source. QC1 and QC2 were samples obtained from an individual subject, while QC3 was composed of a pool of two individual CSF samples. Samples for this purpose were collected retrospectively, and no clinical diagnosis is available for these samples. After their preparation (QC1, 2, and 3), samples were aliquoted and frozen. Before each run, an aliquot was thawed and included in the test run (duplicate testing).

### PET imaging

Of the 50 samples, 49 had PET-Aβ imaging available acquired using either ^11^C-Pittsburgh Compound B (PiB; *N* = 4), ^18^F-florbetapir (FBP; *N* = 15), or ^18^F-flutemetamol (FLUTE; *N* = 30) tracers. The acquisition protocol for each radioligand has been detailed previously [32–34]. Briefly, a 20-min acquisition was started 50 min after either PiB or florbetapir injection and 90 min after flutemetamol injection. Aβ-amyloid PET scans were spatially normalised using CapAIBL [35] and the standard Centiloid (CL) method was applied for quantitation [5]. Participants were classified as PET-Aβ+ if their CL value was 20 or greater; otherwise, they were classed as PET-Aβ−.

### Statistical analysis

Clinical and demographic parameters were assessed via chi-square test (gender, *APOE ε4* allele status), generalised linear modelling (GLM; age, AIBL-PACC), and Kruskal-Wallis test (MMSE, CDR score) where appropriate. Biomarker comparisons between the two extraction methods were conducted using concordance correlations (CC), Passing Block Regression analyses, and Bland-Altman plot analyses. Paired *t*-tests were computed to assess possible differences between biomarker levels between extraction samples, with Box and Whisker plots demonstrating differences in biomarker means between clinical classification for both gravity drip and aspiration samples. Standardised differences between biomarker levels from aspiration and gravity drip extraction methods are presented via error bar plot (Fig. 4). Statistical analyses were performed using the R statistical environment (Version 3.6.1) [36].

## Results

### Cohort demographics

From a total of 50 participants who underwent both gravity drip and aspiration CSF extraction protocols, 49 had PET-Aβ imaging, of which 23 were PET-Aβ+. Of the CN group, 35% were PET-Aβ+, while 71% and 100% of MCI and AD participants respectively were PET-Aβ+. There were no differences in the proportion of males to females, the proportion of *APOE ε4* allele carriage, or age between the three clinical classification groups. As expected, participants with either MCI or AD had significantly lower MMSE scores and significantly higher CDR scores (Table 2).

**Table 2:** Sub-cohort demographics

	Total sample	CN	MCI	AD	*p* value
N (%)	50	36 (72)	8 (16)	6 (12)	
PET-Aβ+ (%)	23 (46)	12 (35)	5 (71)	6 (100)	0.0029
Gender male, *N* (%)	21 (42)	14 (39)	3 (38)	4 (67)	0.54
Mean age, years (SD)	72.8 (5.8)	73.1 (5.6)	73.5 (7.3)	70.5 (5.3)	0.42
*APOE ε4* carriage, *N* (%)	16 (32)	9 (25)	4 (50)	4 (50)	0.26
Median MMSE, (MAD)	28 (3)	29 (1.5)	27 (2.2)	23.5 (4.4)	0.0019
Median CDR score, (MAD)	0 (0)	0 (0)	0.5 (0)	1 (0.4)	< 0.0001

*Abbreviations*: *N* number, *CN* cognitively normal, *MCI* mild cognitive impairment, *AD* Alzheimer’s disease, *APOE ε4* Apolipoprotein E epsilon 4 allele, *APOE ε4* Carriage, *N (%)* number of participants with at least one APOE ε4 allele, *MMSE* Mini-Mental State Examination, *CDR* Clinical Dementia Rating, *IQR* inter-quartile range, *MAD* maximum absolute deviation, *PET-Aβ* Positron Emission Tomography Amyloid Beta. *p* value determined by *t*-test (age), chi-square analyses (PET-Aβ status, *APOE ε4*, and gender) and Kruskal-Wallis tests (CDR score and MMSE)

### Assay characteristics and performance

Table 1 shows the analytical performance characteristics for each biomarker assay. The means for the intra-assay percent coefficient of variation (%CV, standard deviation [SD]) based on duplicate clinical samples were 3.6 (3.0)% for Aβ42, 2.1 (1.79)% for Aβ40, 3.0 (2.6)% for Tau, 3.7 (3.2)% for Neurogranin, 3.4 (2.9)% for α-synuclein, and 3.6 (3.0)% for BACE1.

### Run-acceptance

All kit control concentrations were within the acceptance range for all assays and test runs. OD values for each calibrator concentration were within the standard acceptance criteria. The blank value and highest calibrator point were within specification for all analytes over all test runs. Furthermore, monitoring of the QC samples revealed an inter-assay variability between 5.0% (lowest %CV for Neurogranin) and 14.9% (highest %CV for total Tau; see also results in Table 1).

### Biomarker concordance correlations between CSF extraction methods

**Fig. 1:**
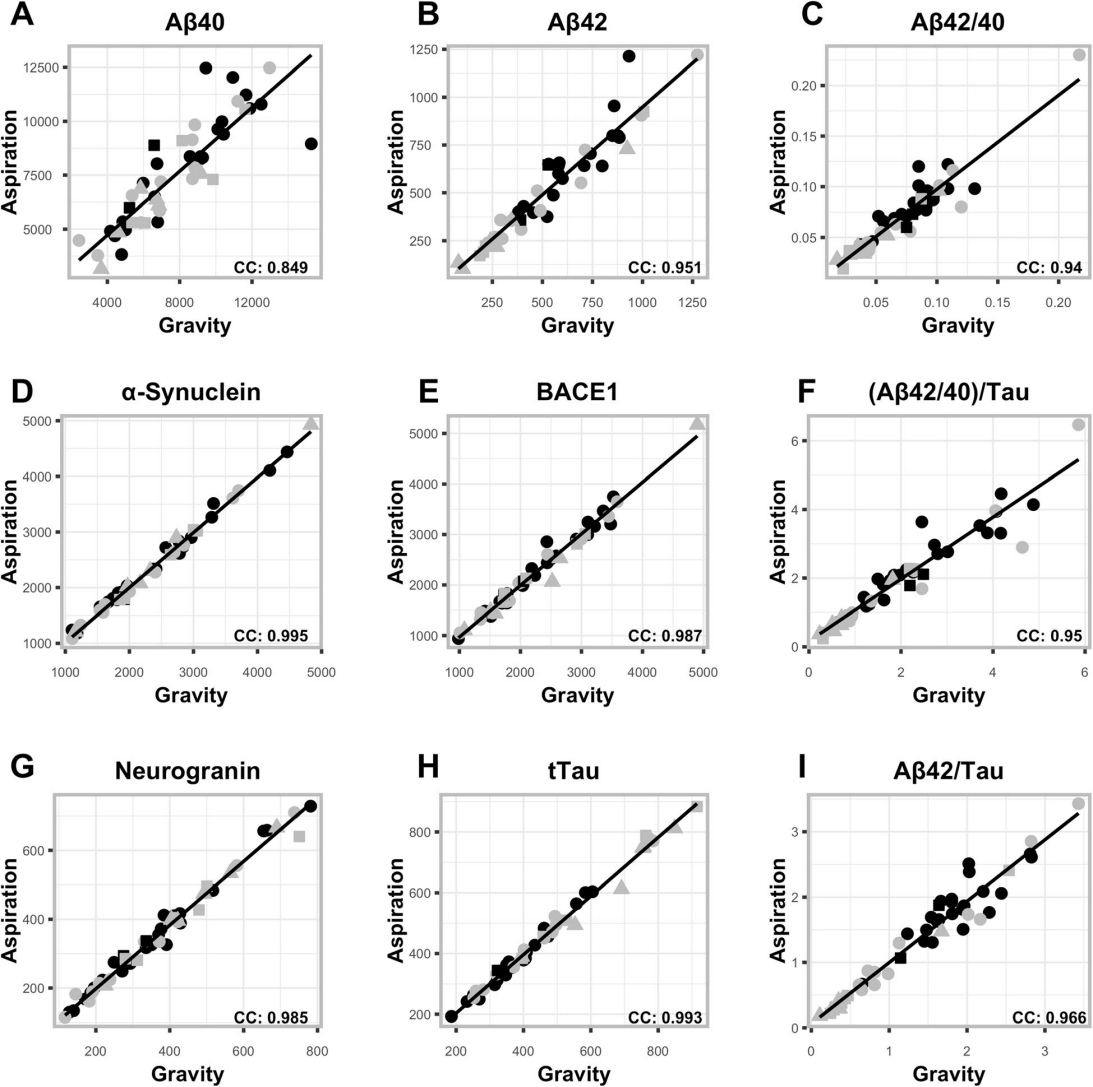
**a**–**i** Concordance correlation plots per biomarker. Black points represent data from positron emission tomography (PET)-Aβ− participants; grey points represent data from PET-Aβ+ participants. Round points represent those participants who were cognitively normal (CN); square points represent those participants with mild cognitive impairment (MCI); triangular points represent those participants with Alzheimer’s disease (AD). The solid black line represents the concordance correlation (CC) between gravity and aspiration extraction methods. Aβ; amyloid beta, BACE1; beta-secretase 1

Using the six biomarkers that were measured, along with the three ratios (Aβ42/40, Aβ42/Tau, and (Aβ42/40)/Tau) for all 50 participants, concordance correlations (CC) were all greater than 0.85 (Fig. 1). Strongest concordance correlations (CC > 0.95) for individual biomarkers between extraction methods were found for Tau (0.993 [95% confidence interval (CI) 0.988–0.996]), α-synuclein (0.995, [0.991–0.997]), BACE1 (0.987 [0.977–0.992]), Neurogranin (0.985 [0.976–0.991]), and Aβ42 (0.951 [0.915–0.972]). Of the three ratios, Aβ42/Tau had the highest CC (0.966 [0.942–0.981]).

Further testing of the concordance via the Passing Bablock method defined regression equations and plots (Supplementary Figure 1) for the relationship between extraction methods. For each biomarker, the plot shows the linear relationship, with thin bootstrap confidence intervals defining small variation between the extraction methods. For individual biomarkers, BACE1, Tau, α-synuclein, and Neurogranin had the smallest confidence intervals, indicating a close fit between the two measurements, whist for the ratio biomarkers the confidence intervals spread wider, with larger biomarker values indicating an increased variance in the fit amongst the larger ratio values.

### Assessment of concordance via Bland-Altman plots

**Fig. 2:**
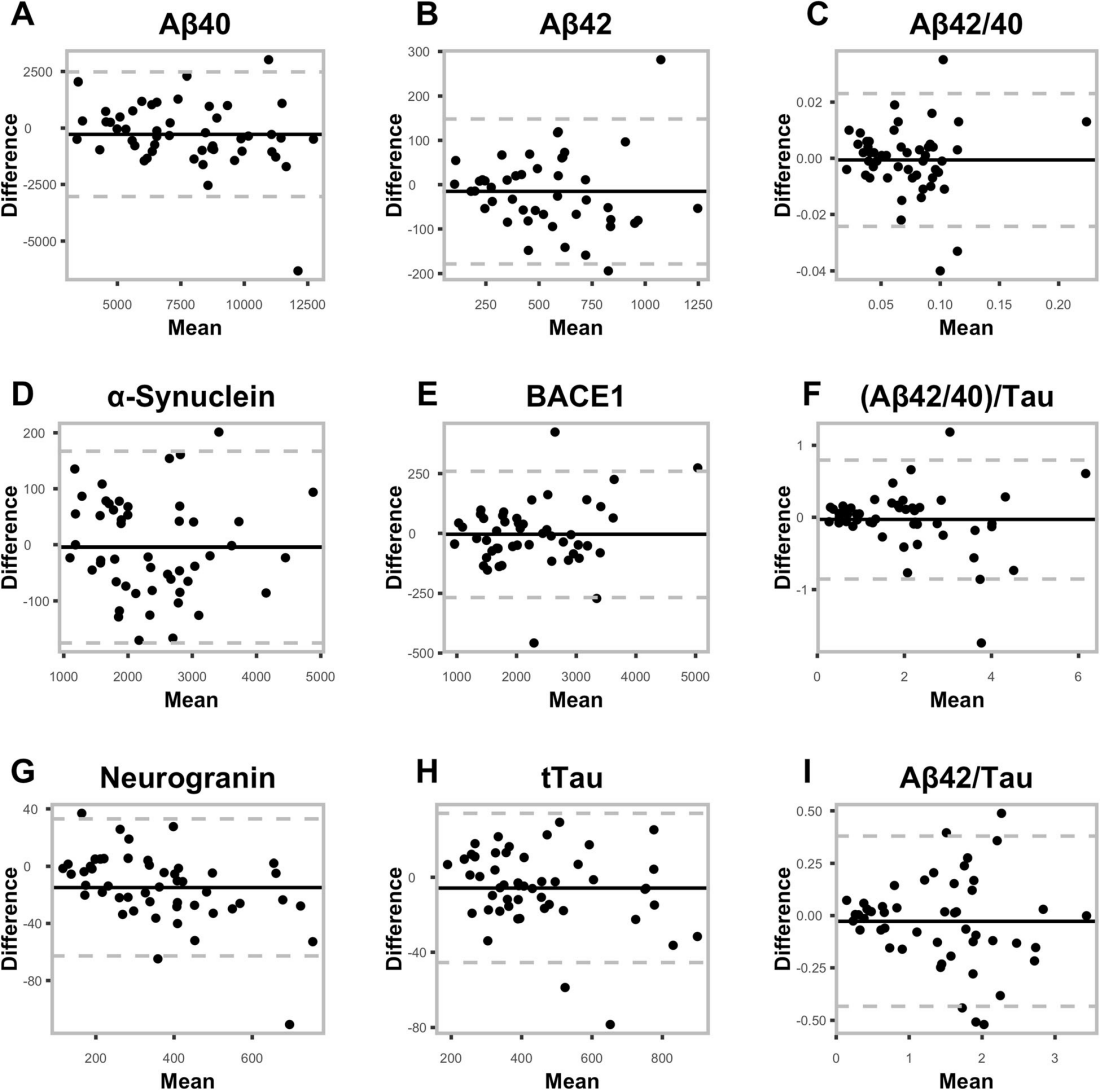
**a**–**i** Bland Altman plots per biomarker. Black horizontal line represents the point at which the biomarker mean difference between aspiration and gravity drip extraction methods is equal to zero. Lower grey dashed line represents the point at which the value on the *y*-axis is 1.96 standard deviations below zero. Upper grey horizontal dashed line represents the point at which the value on the *y*-axis is 1.96 standard deviations above zero

Each of the nine biomarkers (six individual biomarkers and three biomarker ratios) showed a reasonable spread of data points, with plots showing markers having only either one, two, or three points that fit outside ±1.96SD around zero (Fig. 2). Disregarding the outliers, symmetry around the zero-difference line for each individual biomarker was maintained. Lower mean levels for the ratio biomarkers (Aβ42/40)/Tau and Aβ42/Tau demonstrated smaller differences between extraction methods, while larger mean differences showed larger spread of the data. Of the outliers, six participant samples were responsible for all values outside the ± 1.96SD lines. The participant with a large negative difference for BACE1 (− 457.7) also had a large negative difference for Tau (− 78.4). Another participant with unusually high BACE1 values (> 4500) also had a large difference for Tau (− 58.8). The sample with the largest negative difference for Aβ42/40 (− 0.04) also had the largest negative difference for (Aβ42/40)/Tau (− 1.742).

### Paired sample comparisons

Comparing biomarkers via both the complete sample and stratified by clinical classification showed no significant difference in mean biomarker levels between gravity drip and aspiration extraction methods (*p* > 0.05, Table 3). For the individual biomarkers, BACE1 and total Tau had *p* values closer to one (*p* > 0.9), indicating smaller differences between extraction methods, while all ratio biomarkers achieved similar performance (*p* > 0.9). Similar to stratification by clinical classification, there were no statistical differences in biomarker levels found when stratifying data by PET-Aβ status (Supplementary Figure 2).

**Table 3:** Mean values of AD CSF biomarkers, gravity drip versus aspiration extraction method

	All participants *N* = 50			Cognitively normal *N* = 36			Mild cognitive impairment *N* = 8			Alzheimer’s disease *N* = 6		
	Mean (SD)			Mean (SD)			Mean (SD)			Mean (SD)		
	Aspiration	Gravity	*p* value	Aspiration	Gravity	*p* value	Aspiration	Gravity	*p* value	Aspiration	Gravity	*p* value
α-synuclein	2370 (861)	2370 (866)	1.00	2330 (850)	2330 (866)	1.00	2370 (607)	2430 (594)	0.85	2570 (1280)	2530 (1240)	0.95
Neurogranin	356 (162)	371 (174)	0.73	331 (166)	343 (174)	0.77	400 (126)	425 (161)	0.73	446 (154)	466 (158)	0.83
Aβ42	535 (265)	550 (274)	0.88	603 (242)	617 (244)	0.81	405 (263)	416 (274)	0.94	298 (229)	327 (309)	0.86
Aβ40	7570 (2400)	7850 (2800)	0.78	7840 (2510)	8120 (2940)	0.66	7670 (1990)	7900 (2460)	0.84	5820 (1580)	6140 (1940)	0.76
BACE1	2250 (841)	2260 (813)	0.95	2240 (783)	2240 (765)	0.96	2260 (525)	2270 (571)	0.99	2300 (1500)	2370 (1380)	0.93
Tau	450 (176)	456 (181)	0.90	397 (138)	399 (137)	0.96	552 (214)	558 (221)	0.96	634 (166)	666 (165)	0.75
Aβ42/Aβ40	0.069 (0.026)	0.070 (0.028)	0.98	0.076 (0.023)	0.077 (0.025)	0.87	0.051 (0.022)	0.052 (0.025)	0.91	0.047 (0.025)	0.047 (0.030)	0.98
Ratio/Tau*	1.97 (1.30)	2.00 (1.37)	0.93	2.34 (1.29)	2.38 (1.36)	0.90	1.15 (0.79)	1.21 (0.92)	0.89	0.82 (0.58)	0.75 (0.55)	0.83
Aβ42/Tau	1.39 (0.79)	1.42 (0.82)	0.96	1.64 (0.69)	1.67 (0.71)	0.84	0.92 (0.81)	0.93 (0.81)	0.99	0.51 (0.48)	0.53 (0.57)	0.96

*Abbreviations*: *Aβ* amyloid beta, *AD* Alzheimer’s disease, *CSF* cerebrospinal fluid, *SD* standard deviation, *tTau* total tau, *N* number of participants. All biomarker values are expressed in pg/mL. * Ratio; Aβ42/40

Finally, we evaluated whether the observed differences in biomarker concentrations between extraction methods (gravity drip, aspiration) are affected by the selected biomarker or biomarker combination. Results are presented in Fig. 4. While the overall change in concentration between methods is limited, it is obvious from the figure that the variation between subjects is much lower for total Tau, α-synuclein, and Neurogranin as compared to either Aβ proteins used as a single biomarker or when integrated into a ratio.

### Outlier samples

**Fig. 3:**
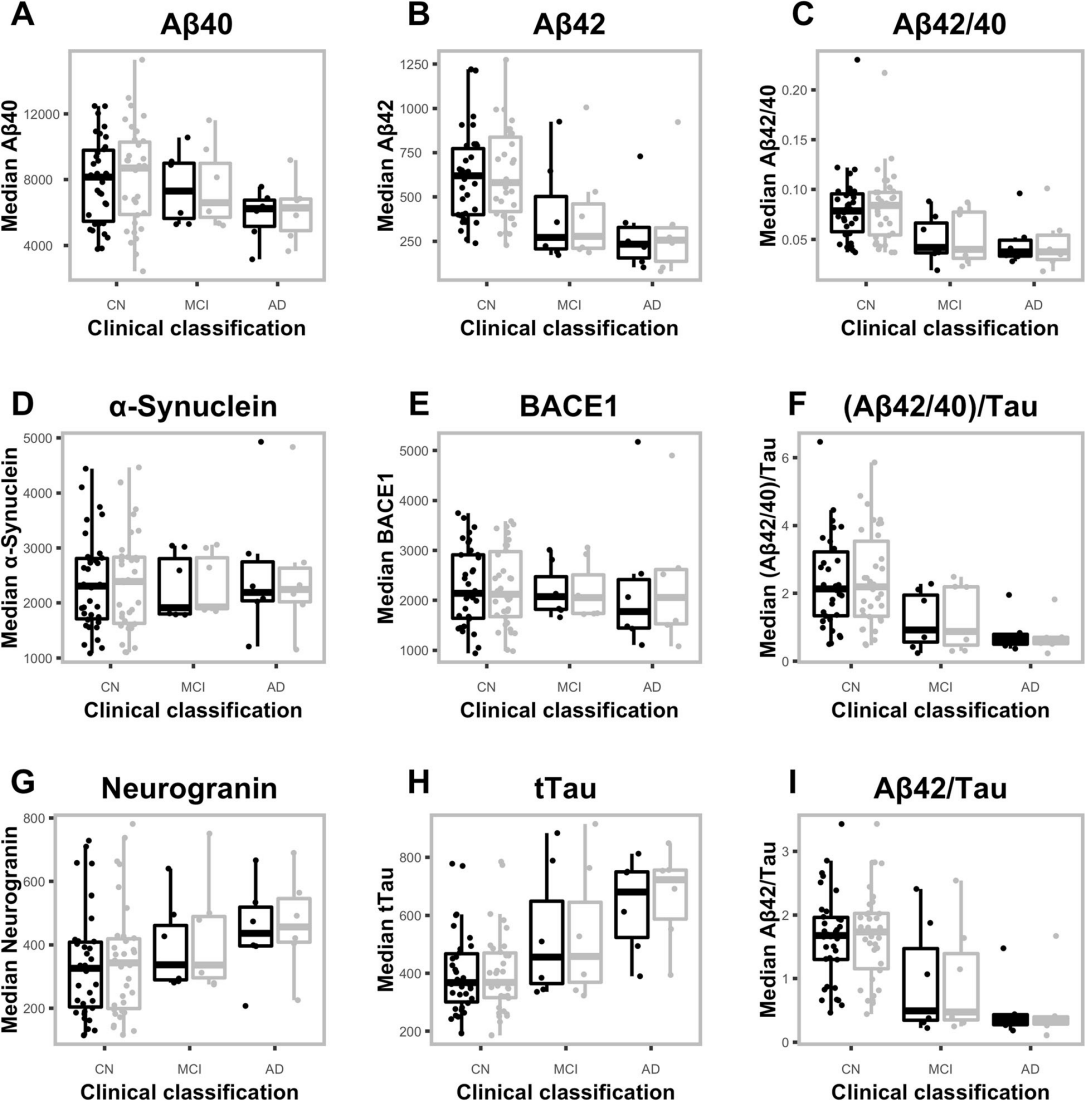
**a**–**i** Box and whisker plots of median biomarker levels between extraction method and clinical classification. Black boxes and points represent data from samples extracted using the aspiration method. Grey boxes and points represent data from samples extracted using the gravity drip method. Upper lines on each box represent the 3rd quartile, middle lines represent the median value, and the lower lines represent the first 1st quartile

Supplementary Table 1 shows the demographic and clinical details for the participants who had CSF values considered as outliers from either gravity drip or aspiration extraction methods. Visualisation refers to how the outlier was detected. For example, the term “Box” refers to the outlier being detected from the Box and Whisker plots (Fig. 3), while the term “BA” refers to the outlier being detected from the Bland-Altman plot (Fig. 2). Here the outlier value represents a large difference in the biomarker value between extraction methods (i.e., the value lies outside the grey dashed lines). Outlier values depicted here are also seen in Fig. 3, where outlier values are consistent across extraction methods.

## Discussions

In this study, we aimed to assess the concordance in biomarker levels between gravity drip and aspiration CSF extraction methods. After investigating each of six individual and three ratio biomarkers using multiple concordance methods, it is clear that biomarker reliability is independent of CSF extraction method. For the majority of biomarkers, concordance correlation coefficients were greater than 0.95, with reduction in precision due to a few outliers and the small sample size. Overall repeatability was consistent within both the complete sample and across individual clinical groups.

Assessment of biomarker outliers from both aspiration and gravity drip extracted samples showed that outlier values were independent of extraction method; i.e., the reason for the aberrant biomarker level was unrelated to extraction method. Furthermore, assessment of biomarker levels between extraction methods, stratified by either clinical classification or by PET-Aβ status, did not increase the variance in biomarker levels, strengthening the claim of stability across a range of different clinical or phenotypic populations.

**Fig. 4:**
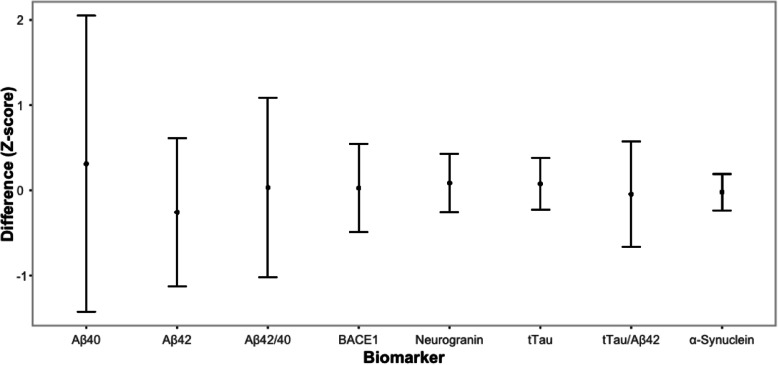
Error bar plot for the standardised difference in biomarker levels between extraction methods. Biomarker values were standardised by removing the mean from each value and then dividing by the standard deviation. Normalised values of biomarkers tested from the gravity drip extraction method were then removed from the values of biomarkers from the aspiration method for plotting. Error bars represent the difference values from each participant, with the mid-point being the point between the minimum and maximum difference values

The minimal changes across extraction methods seen in most biomarkers, as compared to the variability between extraction methods for Aβ40 and Aβ42 (Fig. 4), again reiterates the fact that Aβ is a more challenging protein for analytical assays. The number of confounding factors at the pre-analytical and analytical level that affect Aβ levels in biological fluids is higher than for other proteins. It is not clear precisely which confounding factor(s) might contribute to the variance observed in the current study. No gradient effect was observed in CSF by Le Bastard et al. (2015) for Aβ42, total tau, and pTau181P as measured by ELISA (*n* = 20) [37]. Some gradient effect was seen with higher collection volumes for CSF Aβ42 when analysed on an automated chemiluminesent platform [38]. The same paper provided evidence that CSF-Aβ42 levels were stable if fresh samples were processed within 2 h, followed by a freeze thaw cycle. All AIBL samples were processed within 1 h post collection. Samples were put on wet ice immediately after collection before further processing. Further, Darrow et al. (2020) also noted an effect from blood contamination, especially in the thawed samples. Nevertheless, in this study blood contamination did not account for the higher variances observed for Aβ40 or Aβ42 levels, as verified by quantification of Hb concentrations in each CSF sample. New experimental designs and testing procedures are required to solve such dilemmas.

The method of CSF collection is an important step in the pre-analytical handling of CSF samples. While some investigators routinely use gravity drip, others use aspiration. Gravity drip has the drawback of unpredictable variation of collection times and may potentially take considerably longer than aspiration, thereby reducing feasibility in busy clinics. In dementia evaluation settings, the longer duration of CSF acquisition can be a particular problem for a patient with memory impairment or dementia since repeated reassurance and explanation may be required. Our results demonstrate that syringe aspiration does not have a significant effect on analyte concentrations and, therefore, should be acceptable and allow predictable and more rapid CSF collection.

This report extends the data of Rembach et al. [17] by utilising optimised assay formats and the inclusion of other biomarker proteins which will become important in future stratification of subjects within the several disease areas of neurodegeneration. Our report shows the strong concordance of CSF biomarkers when CSF is collected either by standard gravity drip or syringe aspiration (Table 3). In particular, more rapid collection by aspiration suggests that wider adoption of aspiration is feasible and may become the preferred means of CSF collection for the detection of AD CSF profiles. Shorter duration (~ 10 min from gravity drip to ~ 1 min for aspiration) should help increase acceptance both by patients undergoing the procedure and also staff conducting the LP Furthermore, it is our belief that the outcomes from this study will be relevant to data generated on other technology platforms.

### Limitations

The current study is not without limitations. Firstly, regarding the extractions, all aspirations were performed subsequent to gravity drip collection as per AIBL protocol. Although reversal of collection order is thought unlikely to have any effect on biomarker levels, this notion has not been formally tested. A second limitation of this study was the relatively small number of subjects included, with varying number of samples per clinical classification. The small sample size for participants with MCI/AD may over represent the strength of the biomarker concordance between extraction methods, and future research needs to be performed with a larger sample size to specifically test this. The results of this study should therefore be interpreted in the context of these limitations.

## Conclusions

In summary, the current study provides strong evidence that key CSF protein biomarker measurements are not influenced by extraction through either the gravity drip or aspiration method, and that CSF results utilising either method are inter-changeable. Much time can potentially be saved and subject burden reduced using the syringe extraction approach compared to gravity drip. Results of this study should be incorporated into the new consensus guidelines for CSF collection, storage, and analysis of biomarkers.

## Supplementary Information

**Additional file 1: **Supplementary Table 1. Participant CSF value outliers. CN: Cognitively Normal, MCI: Mild Cognitive Impairment, AD: Alzheimer’s Disease, Box: Box and Whisker plot, BA: Bland Altman plot, Scatter: Scatter plot, *APOE*: Apolipoprotein E ε4 allele (−ve: no ε4 alleles, +ve: at least one ε4 allele), Aβ; Amyloid beta, BACE1; Beta-secretase 1, MMSE; Mini mental score equivalent, CDR; Clinical dementia rating. *ID are random letters given to participants to show where specific individuals were seen to have more than one outlier. Visualisation: where outlier was seen in manuscript figure. Values were considered outliers if they were either outside the dotted lines in the Bland Altman plot, or above/below the whisker on the box and whisker plot, or sitting away from the main data group in the scatter plots. Supplementary Figure 1. Passing Bablock regression fits to biomarker data. Abbreviations: Aβ; Amyloid beta, BACE1; Beta-secretase 1. Diagonal lines represent the slope as calculated via the Passing Bablock method. Shaded grey areas represent the 95% confidence interval as calculated via bootstrap resampling. Light grey dashed line represents the identity line between bottom left and top right corners of the graph. Regression equation is shown in the top left for estimation of the conversion of a data point from aspiration to convert to the same scale as the gravity data point. For example, for any one point on the line that represents a result from a gravity drip extraction for the BACE1 biomarker, we would multiply the value by 1.02 and add the value of − 49.87; e.g., 2000 × 1.02 + (− 49.87) = 1990. Supplementary Figure 2: Box and whisker plots of median biomarker levels between extraction methods and PET-Aβ status. Abbreviations: Aβ; Amyloid beta, BACE1; Beta-secretase 1. Black boxes and points represent data from samples extracted using the aspiration method. Grey boxes and points represent data from samples extracted using the gravity drip method. Upper lines on each box represent the 3rd quartile, middle lines represent the median value and the lower lines represent the 1st quartile. All biomarker comparisons between gravity drip and aspiration by PET Aβ status were not significant (*p* > 0.05, data not shown).

## Data Availability

The datasets used and/or analysed during the current study are available from the corresponding author on reasonable request.

## Abbreviations

AD: Alzheimer’s disease
Aβ: Amyloid beta
CN: Cognitively normal
CSF: Cerebrospinal fluid
FDA: Food and Drug Administration
LP: Lumbar puncture
PET: Positron emission tomography

## References

[1] Jack CR, Knopman DS, Jagust WJ, Shaw LM, Aisen PS, Weiner MW, Petersen RC, Trojanowski JQ (2010). Hypothetical model of dynamic biomarkers of the Alzheimer’s pathological cascade. Lancet Neurol.

[2] Sperling RA, Aisen PS, Beckett LA, Bennett DA, Craft S, Fagan AM, Iwatsubo T, Jack CR, Kaye J, Montine TJ, Park DC, Reiman EM, Rowe CC, Siemers E, Stern Y, Yaffe K, Carrillo MC, Thies B, Morrison-Bogorad M, Wagster MV, Phelps CH (2011). Toward defining the preclinical stages of Alzheimer's disease: recommendations from the National Institute on Aging-Alzheimer's Association workgroups on diagnostic guidelines for Alzheimer's disease. Alzheimers Dement.

[3] Jack CR, Bennett DA, Blennow K, Carrillo MC, Dunn B, Haeberlein SB, NIA-AA, et al. (2018). Research framework: toward a biological definition of Alzheimer’s disease. Alzheimers Dement.

[4] Uzuegbunam BC, Librizzi D, Yousefi BH (2020). PET radiopharmaceuticals for Alzheimer’s disease and Parkinson’s disease diagnosis, the current and future landscape. Molecules.

[5] Klunk WE, Koeppe RA, Price JC, Benzinger TL, Devous MD, Jagust WJ, Johnson KA, Mathis CA, Minhas D, Pontecorvo MJ, Rowe CC, Skovronsky DM, Mintun MA (2015). The Centiloid Project: standardizing quantitative amyloid plaque estimation by PET. Alzheimers Dement.

[6] Center for drug evaluation and research. (2011). Clinical Review NDA 202-008 A18. https://www.accessdata.fda.gov/drugsatfda_docs/nda/2012/202008Orig1s000MedR.pdf. Accessed Oct 2020

[7] Vanderstichele HM, Teunissen CE, Vanmechelen E (2019). Critical steps to be taken into consideration before quantification of β-amyloid and tau isoforms in blood can be implemented in a clinical environment. Neurol Ther.

[8] USA Food and Drug Administration. (2020). FDA News Release: FDA approves first drug to image tau pathology in patients being evaluated for Alzheimer’s disease. https://www.fda.gov/news-events/press-announcements/fda-approves-first-drug-image-tau-pathology-patients-being-evaluated-alzheimers-disease. Accessed Oct 2020

[9] Blennow K, Hampel H (2003). CSF markers for incipient Alzheimer’s disease. Lancet Neurol.

[10] Duits FH, Martinez-Lage P, Paquet C, Engelborghs S, Lleó A, Hausner L, Molinuevo JL, Stomrud E, Farotti L, IHGB R, Tsolaki M, Skarsgård C, Åstrand R, Wallin A, Vyhnalek M, Holmber-Clausen M, Forlenza OV, Ghezzi L, Ingelsson M, Hoff EI, Roks G, de Mendonça A, Papma JM, Izagirre A, Taga M, Struyfs H, Alcolea DA, Frölich L, Balasa M, Minthon L, JWR T, Persson S, Zetterberg H, van der Flier WM, Teunissen CE, Scheltens P, Blennow K (2016). Performance and complications of lumbar puncture in memory clinics: Results of the multicenter lumbar puncture feasibility study. Alzheimers Dement.

[11] Shaw LM, Arias J, Blennow K, Galasko D, Molinuevo JL, Salloway S, Schindler S, Carrillo MC, Hendrix JA, Ross A, Illes J, Ramus C, Fifer S (2018). Appropriate use criteria for lumbar puncture and cerebrospinal fluid testing in the diagnosis of Alzheimer’s disease. Alzheimers Dement.

[12] McDade E, Wang G, Gordon BA, Hassenstab J, TLS B, Buckles V, Fagan AM, Holtzman DM, Cairns NJ, Goate AM, Marcus DS, Morris JC, Paumier K, Xiong C, Allegri R, Berman SB, Klunk W, Noble J, Ringman J, Ghetti B, Farlow M, Sperling RA, Chhatwal J, Salloway S, Graff-Radford NR, Schofield PR, Masters C, Rossor MN, Fox NC, Levin J, Jucker M, Bateman RJ, Dominantly Inherited Alzheimer Network (2018). Longitudinal cognitive and biomarker changes in dominantly inherited Alzheimer disease. Neurology.

[13] Palmqvist S, Mattsson N, Hansson O, Alzheimer’s Disease Neuroimaging Initiative (2016). Cerebrospinal fluid analysis detects cerebral amyloid-β accumulation earlier than positron emission tomography. Brain.

[14] Blennow K, Hampel H, Weiner M, Zetterberg H (2010). Cerebrospinal fluid and plasma biomarkers in Alzheimer disease. Nat Rev Neurol.

[15] Molinuevo JL, Ayton S, Batrla R, Bednar MM, Bittner T, Cummings J, Fagan AM, Hampel H, Mielke MM, Mikulskis A, O'Bryant S, Scheltens P, Sevigny J, Shaw LM, Soares HD, Tong G, Trojanowski JQ, Zetterberg H, Blennow K (2018). Current state of Alzheimer’s fluid biomarkers. Acta Neuropathol.

[16] European Medicines Agency. Qualification opinion of Alzheimer’s disease novel methodologies/biomarkers for the use of CSF Aβ1–42 and t-tau and/or PET-amyloid imaging (positive/ negative) as biomarkers for enrichment, for use in regulatory clinical trials in mild and moderate Alzheimer’s disease*.*. https://www.ema.europa.eu/en/documents/regulatory-procedural-guideline/qualification-opinion-alzheimers-disease-novel-methodologies/biomarkers-use-cerebrospinal-fluid-amyloid-beta-1-42-t-tau/positron-emission-tomography-amyloid-imaging-positive/negative_en.pdf. Accessed Oct 2020

[17] USA Food and Drug Administration (2020). Drug Development Tool (DDT) Qualification Programs.

[18] Hansson O, Mikulskis A, Fagan AM, Teunissen C, Zetterberg H, Vanderstichele H, Molinuevo JL, Shaw LM, Vandijck M, Verbeek MM, Savage M, Mattsson N, Lewczuk P, Batrla R, Rutz S, Dean RA, Blennow K (2018). The impact of preanalytical variables on measuring cerebrospinal fluid biomarkers for Alzheimer’s disease diagnosis: a review. Alzheimers Dement.

[19] Mattsson N, Andreasson U, Persson S, Carrillo MC, Collins S, Chalbot S, Cutler N, Dufour-Rainfray D, Fagan AM, Heegaard NH, Robin Hsiung GY, Hyman B, Iqbal K, Kaeser SA, Lachno DR, Lleó A, Lewczuk P, Molinuevo JL, Parchi P, Regeniter A, Rissman RA, Rosenmann H, Sancesario G, Schröder J, Shaw LM, Teunissen CE, Trojanowski JQ, Vanderstichele H, Vandijck M, Verbeek MM, Zetterberg H, Blennow K, Alzheimer’s Association QC Program Work Group (2013). CSF biomarker variability in the Alzheimer’s Association quality control program. Alzheimers Dement.

[20] Boulo S, Kuhlmann J, Andreasson U, Brix B, Venkataraman I, Herbst V, Rutz S, Manuilova E, Vandijck M, Dekeyser F, Bjerke M, Pannee J, Charoud-Got J, Auclair G, Mazoua S, Pinski G, Trapmann S, Schimmel H, Emons H, Quaglia M, Portelius E, Korecka M, Shaw LM, Lame M, Chambers E, Vanderstichele H, Stoops E, Leinenbach A, Bittner T, Jenkins RG, Kostanjevecki V, Lewczuk P, Gobom J, Zetterberg H, Zegers I, Blennow K (2020). First amyloid β1–42 certified reference material for re-calibrating commercial immunoassays. Alzheimers Dement.

[21] Blennow K, Zetterberg H (2019). Fluid biomarker-based molecular phenotyping of Alzheimer’s disease patients in research and clinical settings. Prog Mol Biol Transl Sci.

[22] Vanderstichele H, Bibl B, Engelborghs S, Le Bastard N, Lewczuk P, Molinuevo JL, Parnetti L, Perret-Liaudet A, Shaw LM, Teunissen C, Wouters D, Blennow K (2012). Standardization of preanalytical aspects of cerebrospinal fluid biomarker testing for Alzheimer’s disease diagnosis: a consensus paper from the Alzheimer’s Biomarkers Standardization Initiative. Alzheimers Dement.

[23] del Campo M, Mollenhauer B, Bertolotto A, Engelborghs S, Hampel H, Simonsen AH, Kapaki E, Kruse N, Le Bastard N, Lehmann S, Molinuevo JL, Parnetti L, Perret-Liaudet A, Sáez-Valero J, Saka E, Urbani A, Vanmechelen E, Verbeek M, Visser PJ, Teunissen C (2012). Recommendations to standardize preanalytical confounding factors in Alzheimer’s and Parkinson’s disease cerebrospinal fluid biomarkers: an update. Biomark Med.

[24] Hansson O, Batrla R, Brix B, Carrillo MC, Corradini V, Edelmayer RM, Esquivel RN, Hall C, Lawson J, Bastard NL, Molinuevo JL, Nisenbaum LK, Rutz S, Salamone SJ, Teunissen C, Traynham C, Umek RM, Vanderstichele H, Vandijck M, Wahl S, Weber CJ, Zetterberg H, and Blennow K. The Alzheimer’s association international guidelines for handling of cerebrospinal fluid for routine clinical measurements of amyloid β and tau. Alz Dementia (Submitted). 10.1002/alz.12316 33788410

[25] Fagan AM, Shaw LM, Xiong C, Vanderstichele H, Mintun HA, Trojanowski JQ, Coart E, Morris JC, Holtzman DM (2011). Comparison of analytical platforms for cerebrospinal fluid measures of beta-amyloid 1-42, total tau, and p-tau181 for identifying Alzheimer disease amyloid plaque pathology. Arch Neurol.

[26] Jurado R and Walker K. (1990). Clinical methods: the history, physical, and laboratory examinations. 3rd edition. Chapter 74 cerebrospinal fluid. 21250045

[27] Ellis KA, Bush AI, Darby D, De Fazio D, Foster J, Hudson P, Lautenschlager NT, Lenzo N, Martins RN, Maruff P, Masters C, Milner A, Pike K, Rowe C, Savage G, Szoeke C, Taddei K, Villemagne V, Woodward M, Ames D, AIBL Research Group (2009). The Australian imaging, biomarkers and lifestyle (AIBL) study of aging: methodology and baseline characteristics of 1112 individuals recruited for a longitudinal study of Alzheimer’s disease. Int Psychogeriatr.

[28] Rembach A, Evered LA, Li QX, Nash T, Vidaurre L, Fowler CJ, Pertile KK, Rumble RL, Trounson BO, Maher S, Mooney F, Farrow M, Taddei K, Rainey-Smith S, Laws SM, Macaulay SL, Wilson W, Darby DG, Martins RN, Ames D, Collins S, Silbert B, Masters CL, Doecke JD, AIBL Research Group (2015). Alzheimer’s disease cerebrospinal fluid biomarkers are not influenced by gravity drip or aspiration extraction methodology. Alzheimers Res Ther.

[29] Paciottia S, Stoops E, Françoisc C, Bellomob G, Eusebid P, Vanderstichele H, Chiasserinia D, Parnetti L. Cerebrospinal fluid hemoglobin levels as markers of blood contamination: relevance for α-synuclein measurement. (Submitted & in review). 10.1515/cclm-2020-1521 33957709

[30] Mollenhauer B, Batrla R, El-Agnaf O, Galasko DR, Lashuel HA, Merchant KM, Shaw LM, Selkoe DJ, Umek R, Vanderstichele H, Zetterberg H, Zhang J, Caspell-Garcia C, Coffey C, Hutten SJ, Frasier M, Taylor P, Investigating Synuclein Consortium of the Michael J. Fox Foundation for Parkinson’s Research (2017). A user’s guide for α-synuclein biomarker studies in biological fluids: Perianalytical considerations. Mov Disord.

[31] Vanderstichele HM, Janelidze S, Demeyer L, Coart E, Stoops E, Herbst V, Mauroo K, Brix B, Hansson O (2016). Optimized standard operating procedures for the analysis of cerebrospinal fluid Aβ42 and the ratios of Aβ isoforms using low protein binding tubes. J Alzheimers Dis.

[32] Rowe CC, Ellis KA, Rimajova M, Bourgeat P, Pike KE, Jones G, Fripp J, Tochon-Danguy H, Morandeau L, O’Keefe G, Price R, Raniga P, Robins P, Acosta O, Lenzo N, Szoeke C, Salvado O, Head R, Martins RM, C.L., Ames D, Villemagne V. (2010). Amyloid imaging results from the Australian imaging, biomarkers and lifestyle (AIBL) study of aging. Neurobiol Aging.

[33] Wong DF, Rosenberg PB, Zhou Y, Kumar A, Raymont V, Ravert HT, Dannals RF, Nandi A, Brasi’C JR, Ye W, Hilton J, Lyketsos C, Kung HF, Joshi AD, Skovronsky DM, Pontecorvo MJ. In vivo imaging of amyloid deposition in Alzheimer disease using the radioligand 18F-AV-45 (florbetapir [corrected] F 18). J Nucl Med 2010;51, 913–920, 6, doi: 10.2967/jnumed.109.069088. 10.2967/jnumed.109.069088 PMC3101877 20501908

[34] Vandenberghe R, Van Laere K, Ivanoiu A, Salmon E, Bastin C, Triau E, Hasselbalch S, Law I, Andersen A, Korner A, Minthon L, Garraux G, Nelissen N, Bormans G, Buckley C, Owenius R, Thurfjell L, Farrar G, Brooks DJ (2010). 18Fflutemetamol amyloid imaging in Alzheimer disease and mild cognitive impairment: a phase 2 trial. Ann Neurol.

[35] Bourgeat P, Doré V, Fripp J, Ames D, Masters CL, Salvado O, Villemagne VL, Rowe CC (2018). Implementing the centiloid transformation for 11c-PiB and β-amyloid 18f-PET tracers using CapAIBL. NeuroImage..

[36] Core Team R (2020). R: a language and environment for statistical computing.

[37] Le Bastard N, De Deyn PP, Engelborghs S (2015). Importance and impact of preanalytical variables on Alzheimer disease biomarker concentrations in cerebrospinal fluid. Clin Chem.

[38] Jacqueline A Darrow, Amanda Calabro, Sara Gannon, Amanze Orusakwe, Rianne Esquivel, C J Traynham, Aruna Rao, Seema Gulyani, Kristina Khingelova, Karen Bandeen-Roche, Marilyn Albert, Abhay Moghekar. Effect of patient-specific preanalytic variables on CSF Aβ1–42 concentrations measured on an automated chemiluminescent platform, J Appl Laboratory Med. 2020;ePub Ahead of print. 10.1093/jalm/jfaa145 PMC8482291 33249440

