# Supplementary material for: Core Alzheimer’s disease cerebrospinal fluid biomarker assays are not affected by aspiration or gravity drip extraction methods
Source: Alzheimers Res Ther. 2021 Apr 16;13:79. doi: 10.1186/s13195-021-00812-9 (PMC8052760; doi:10.1186/s13195-021-00812-9)
Supplement: Supplementary file 1 — Additional file 1: Supplementary Table 1. Participant CSF value outliers. CN: Cognitively Normal, MCI: Mild Cognitive Impairment, AD: Alzheimer’s Disease, Box: Box and Whisker plot, BA: Bland Altman plot, Scatter: Scatter plot, APOE: Apolipoprotein E ε4 allele (−ve: no ε4 alleles, +ve: at least one ε4 allele), Aβ; Amyloid beta, BACE1; Beta-secretase 1, MMSE; Mini mental score equivalent, CDR; Clinical dementia rating. *ID are random letters given to participants to show where specific individuals were seen to have more than one outlier. Visualisation: where outlier was seen in manuscript figure. Values were considered outliers if they were either outside the dotted lines in the Bland Altman plot, or above/below the whisker on the box and whisker plot, or sitting away from the main data group in the scatter plots. Supplementary Figure 1. Passing Bablock regression fits to biomarker data. Abbreviations: Aβ; Amyloid beta, BACE1; Beta-secretase 1. Diagonal lines represent the slope as calculated via the Passing Bablock method. Shaded grey areas represent the 95% confidence interval as calculated via bootstrap resampling. Light grey dashed line represents the identity line between bottom left and top right corners of the graph. Regression equation is shown in the top left for estimation of the conversion of a data point from aspiration to convert to the same scale as the gravity data point. For example, for any one point on the line that represents a result from a gravity drip extraction for the BACE1 biomarker, we would multiply the value by 1.02 and add the value of − 49.87; e.g., 2000 × 1.02 + (− 49.87) = 1990. Supplementary Figure 2: Box and whisker plots of median biomarker levels between extraction methods and PET-Aβ status. Abbreviations: Aβ; Amyloid beta, BACE1; Beta-secretase 1. Black boxes and points represent data from samples extracted using the aspiration method. Grey boxes and points represent data from samples extracted using the gravity drip method. Upper [file 13195_2021_812_MOESM1_ESM.docx]

**Supplementary materials**

| Biomarker | Method | Value | Visualisation | ID* | Classification | PET-Aβ | Age | *APOE ε4* | MMSE | CDR |
| --- | --- | --- | --- | --- | --- | --- | --- | --- | --- | --- |
| α−synuclein | Aspiration |  | Box |  | AD | Aβ+ | 65 | non-ε4 | 27 | 0.5 |
|  | Gravity |  | Box |  | AD | Aβ+ | 65 | non-ε4 | 27 | 0.5 |
|  | Aspiration | 4461 | Box | B | CN | Aβ- | 85 | non-ε4 | 28 | 0 |
|  | Gravity | 4439 | Box | B | CN | Aβ- | 85 | non-ε4 | 28 | 0 |
| Neurogranin | Aspiration | 728.5 | Box | C | CN | Aβ- | 77 | non-ε4 | 29 | 0 |
|  | Gravity | 781.3 | Box | C | CN | Aβ- | 77 | non-ε4 | 29 | 0 |
|  | Aspiration | 640.2 | Box/BA | D | MCI | Aβ+ | 77 | non-ε4 | 27 | 0.5 |
|  | Gravity | 750.8 | Box/BA | D | MCI | Aβ+ | 77 | non-ε4 | 27 | 0.5 |
| Aβ40 | Aspiration | 8954 | Box/BA | E | CN | Aβ- | 73 | non-ε4 | 26 | 0 |
|  | Gravity | 15278 | Box/BA | E | CN | Aβ- | 73 | non-ε4 | 26 | 0 |
| Aβ42 | Aspiration | 1220 | Box/Scatter | F | CN | Aβ+ | 81 | non-ε4 | 26 | 0 |
|  | Gravity | 1274 | Box/Scatter | F | CN | Aβ+ | 81 | non-ε4 | 26 | 0 |
|  | Aspiration | 925.5 | Box | G | MCI | Aβ+ | 77 | non-ε4 | 30 | 0.5 |
|  | Gravity | 1006 | Box | G | MCI | Aβ+ | 77 | non-ε4 | 30 | 0.5 |
|  | Aspiration | 729.2 | Box | A | AD | Aβ+ | 65 | non-ε4 | 27 | 0.5 |
|  | Gravity | 923.2 | Box | A | AD | Aβ+ | 65 | non-ε4 | 27 | 0.5 |
|  | Aspiration | 1214 | BA | B | CN | Aβ- | 85 | non-ε4 | 28 | 0 |
|  | Gravity | 932.7 | BA | B | CN | Aβ- | 85 | non-ε4 | 28 | 0 |
| BACE1 | Aspiration | 5172 | Box/Scatter | A | AD | Aβ+ | 65 | non-ε4 | 27 | 0.5 |
|  | Gravity | 4898 | Box/Scatter | A | AD | Aβ+ | 65 | non-ε4 | 27 | 0.5 |
|  | Aspiration | 2063 | BA | H | AD | Aβ+ | 63 | non-ε4 | 26 | 0.5 |
|  | Gravity | 2520 | BA | H | AD | Aβ+ | 63 | non-ε4 | 26 | 0.5 |
|  | Aspiration | 2856 | BA | I | CN | Aβ- | 79 | non-ε4 | 28 | 0 |
|  | Gravity | 2432 | BA | I | CN | Aβ- | 79 | non-ε4 | 28 | 0 |
| Tau | Aspiration | 777.8 | Box | J | CN | Aβ+ | 71 | non-ε4 | 30 | 0 |
|  | Gravity | 773.5 | Box | J | CN | Aβ+ | 71 | non-ε4 | 30 | 0 |
|  | Aspiration | 770.1 | Box | K | CN | Aβ+ | 67 | ε4+ | 30 | 0.5 |
|  | Gravity | 784.9 | Box | K | CN | Aβ+ | 67 | ε4+ | 30 | 0.5 |
|  | Aspiration | 612.7 | BA | H | AD | Aβ+ | 63 | non-ε4 | 26 | 0.5 |
|  | Gravity | 691.1 | BA | H | AD | Aβ+ | 63 | non-ε4 | 26 | 0.5 |
|  | Aspiration | 494.1 | BA |  | AD | Aβ+ | 65 | non-ε4 | 27 | 0.5 |
|  | Gravity | 552.9 | BA |  | AD | Aβ+ | 65 | non-ε4 | 27 | 0.5 |

**Supplementary Table 1.** Participant CSF value outliers. CN: Cognitively Normal, MCI: Mild Cognitive Impairment, AD: Alzheimer’s Disease, Box: Box and Whisker plot, BA: Bland Altman plot, Scatter: Scatter plot, *APOE*: Apolipoprotein E ε4 allele (-ve: no ε4 alleles, +ve: at least one ε4 allele), A Amyloid beta, BACE1; Beta-secretase 1, MMSE; Mini mental score equivalent, CDR; Clinical dementia rating. *ID are random letters given to participants to show where specific individuals were seen to have more than one outlier. Visualisation: where outlier was seen in manuscript figure. Values were considered outliers if they were either outside the dotted lines in the Bland Altman plot, or above/below the whisker on the box and whisker plot, or sitting away from the main data group in the scatter plots.

**Supplementary Figure 1**


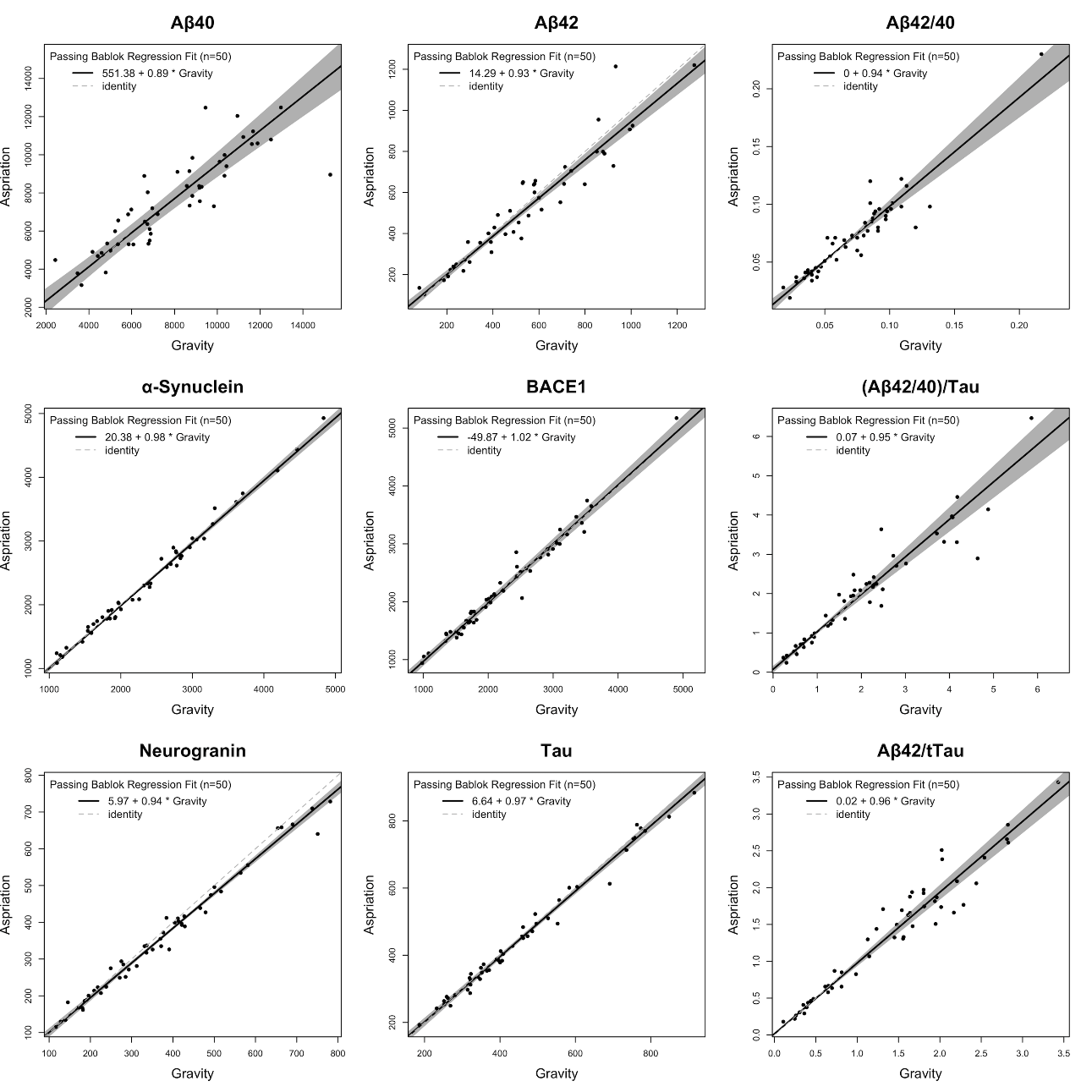


**Supplementary Figure 1: Passing Bablock regression fits to biomarker data.**

Abbreviations: A Amyloid beta, BACE1; Beta-secretase 1. Diagonal lines represent the slope as calculated via the Passing Bablock method. Shaded grey areas represent the 95% confidence interval as calculated via bootstrap resampling. Light grey dashed line represents the identity line between bottom left and top right corners of the graph. Regression equation is shown in the top left for estimation of the conversion of a data point from aspiration to convert to the same scale as the gravity data point. For example, for any one point on the line that represents a result from a gravity drip extraction for the BACE1 biomarker, we would multiply the value by 1.02 and add the value of -49.87; e.g., 2000 x 1.02 + (-49.87) = 1990.

**Supplementary Figure 2**


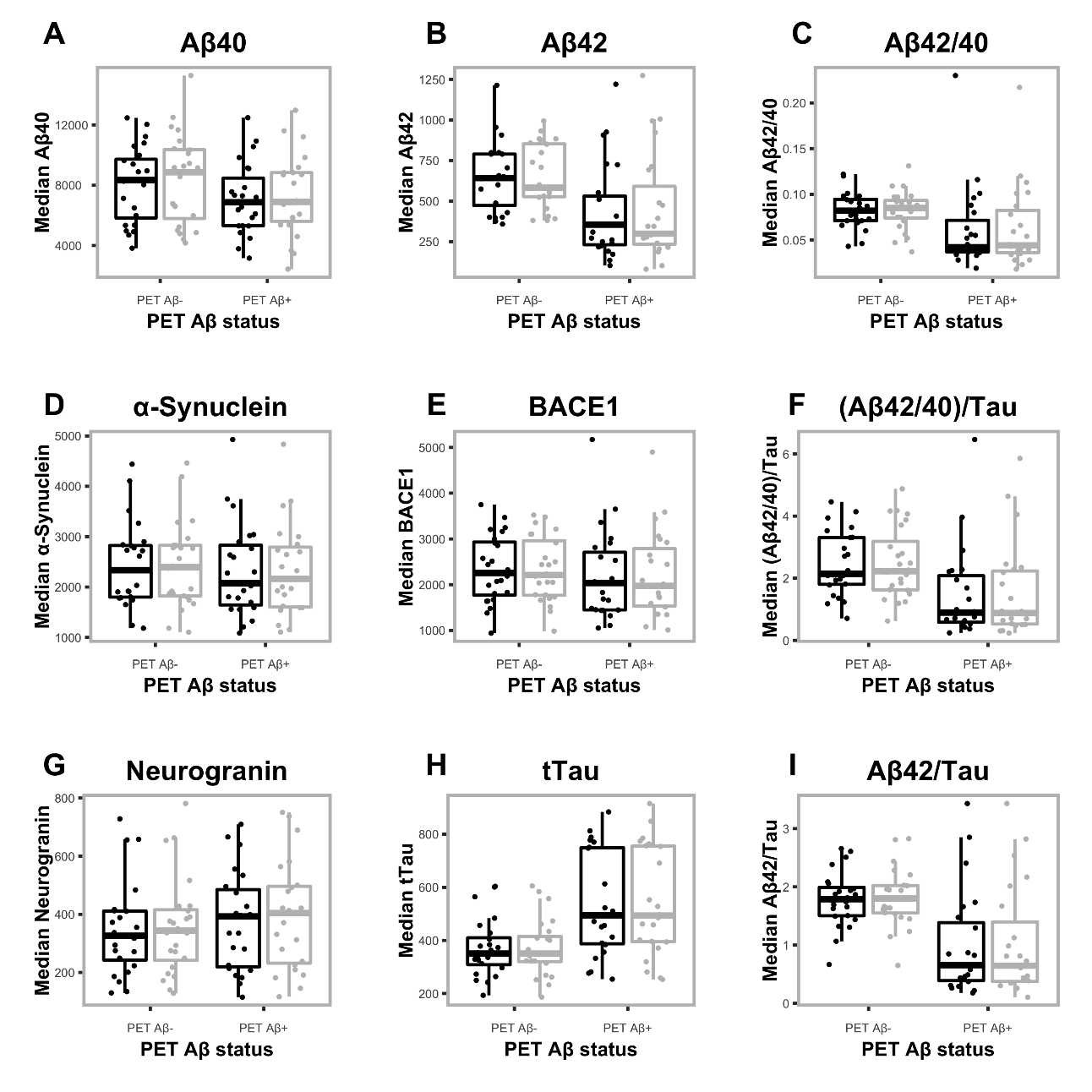

**Supplementary Figure 2: Box and whisker plots of median biomarker levels between extraction methods and PET-Aβ status.**

Abbreviations: A Amyloid beta, BACE1; Beta-secretase 1. Black boxes and points represent data from samples extracted using the aspiration method. Grey boxes and points represent data from samples extracted using the gravity drip method. Upper lines on each box represent the 3^rd^ quartile, middle lines represent the median value and the lower lines represent the 1^st^ quartile. All biomarker comparisons between gravity drip and aspiration by PET Aβ status were not significant (p >0.05, data not shown).
